# Supplementary material for: An ultrawide-range photochromic molecular fluorescence emitter
Source: Nat Commun. 2024 Jun 26;15:5401. doi: 10.1038/s41467-024-49670-7 (PMC11208420; doi:10.1038/s41467-024-49670-7)
Supplement: Supplementary file 1 — Supplementary Information [file 41467_2024_49670_MOESM1_ESM.pdf]

Supplementary Information

An ultrawide-range photochromic molecular fluorescence emitter

Xiao Chen,[1] Xiao-Fang Hou,[1] Xu-Man Chen,[1,*] and Quan Li[1,2,*]

[1]Institute of Advanced Materials and School of Chemistry and Chemical Engineering, Southeast University, Nanjing 211189, China
*E-mail: chenxm@seu.edu.cn, quanli3273@gmail.com

[2]Materials Science Graduate Program, Kent State University, Kent, OH 44242, USA

Table of Contents

A. **Supplementary Methods**

B. **Supplementary Notes 1.** Photophysical properties of PMC and PSP.

C. **Supplementary Notes 2.** Investigation of ICT and self-assembly behavior of PMC.

D. **Supplementary Notes 3.** Solvent effect of photochromic fluorescence of PMC.

E. **Supplementary Notes 4.** Photochromic fluorescence of PMC in PMMA film.

F. **Supplementary Figures.**

A. Supplementary Methods

Materials: Chemical reagents are purchased from commercial resource and used without further purification. 2,3,3-Trimethylindolenine is purchased from Aladdin Reagent (Shanghai) Co., Ltd. Propane sultone is purchased from Energy chemical Co., Ltd., 2-hydroxybenzaldehyde and 1-pyrenylboronic acid are purchased from Bidepharm. $Pd(PPh_3)_4$ is purchased from Leyan. Other solvents are purchased from Sinopharm Chemical Reagent Co., Ltd.

Synthesis of 2-hydroxy-4-(pyren-1-yl)benzaldehyde (1): Under N_2 protection, 2-hydroxybenzaldehyde (1.50 g, 7.46 mmol), 1-pyrenylboronic acid (1.5 eq), $Pd(PPh_3)_4$ (0.015 eq) and K_2CO_3 (3.0 eq) were dissolved in 40 mL 1,4-Dioxane/H_2O (4:1) in a Schlenk flask. The reaction mixture was heated to 85°C for 24 hours. After the reaction, the mixture was extracted with ethyl acetate three times and organic layer was concentrated under vacuum. The obtained crude product was then purified by flash chromatography with gradient elution (from 5% ethyl acetate in petroleum ether to 50% ethyl acetate in petroleum ether in 45 mins) to give a yellow solid (1.5 g, yield: 62.5%). ^{1}H-NMR (600 MHz, DMSO-d_6) δ: 11.00 (s, 1H), 10.41 (s, 1H), 8.43 – 8.31 (m, 3H), 8.26 (s, 2H), 8.22 (d, J = 9.2 Hz, 3H), 8.16 – 8.10 (m, 2H), 8.04 (d, J = 7.8 Hz, 1H), 7.88 (d, J = 7.9 Hz, 1H), 7.30 – 7.19 (m, 2H). ^{13}C NMR (151 MHz, DMSO-d_6) δ: 190.16, 160.62, 148.84, 136.21, 131.40, 131.13, 130.80, 129.64, 128.59, 128.33, 127.92, 127.81, 127.61, 127.05, 126.14, 125.77, 125.46, 124.75, 124.54, 124.40, 122.72, 121.92, 119.38. HRMS (m/z)： [M-H]$^-$ calcd. for: $C_{23}H_{13}O_2$, 321.09101, found, 321.09213.

Synthesis of 2,3,3-trimethyl-1-(3-sulfonatepropyl)-3H-indolium (2): The 2 is synthesized according to the previous work[1]. That is: Under N_2 protection, 2,3,3-trimethylindolenine (10 g, 62.8 mmol) was firstly added into 50 mL acetonitrile followed by propane sultone (1.26 g, 1.1 eq). The mixture was stirred at 80 °C overnight. Then, the reaction solution was dropped into ethyl acetate at room temperature. The purple solid was collected by filtration, washed with ethyl acetate and n-hexane three times to obtain 15.2 g product (yield: 91.18%). ^{1}H-NMR (600 MHz, DMSO-d_6): δ: 8.05

(d, 8.4 Hz 1H), 7.82 (dd, J = 7.1, 1.6 Hz, 1H), 7.65-7.50 (m, 1H), 4.76 – 4.53 (t, J = 7. 2 2H), 2.83 (s, 3H), 2.62 (t, J = 6.5 Hz, 2H), 2.15 (td, J = 7.1, 6.6, 3.5 Hz, 2H), 1.53 (s, 6H). ^{13}C NMR (151 MHz, DMSO-d_6) δ 197.02, 142.41, 141.68, 129.80, 129.43, 123.91, 115.93, 54.60, 47.84, 47.05, 24.23, 22.52, 14.27. HRMS (m/z)： $[M+H]^+$ calcd. for: $C_{14}H_{19}NO_3S$, 282.11584, found, 282.11510.

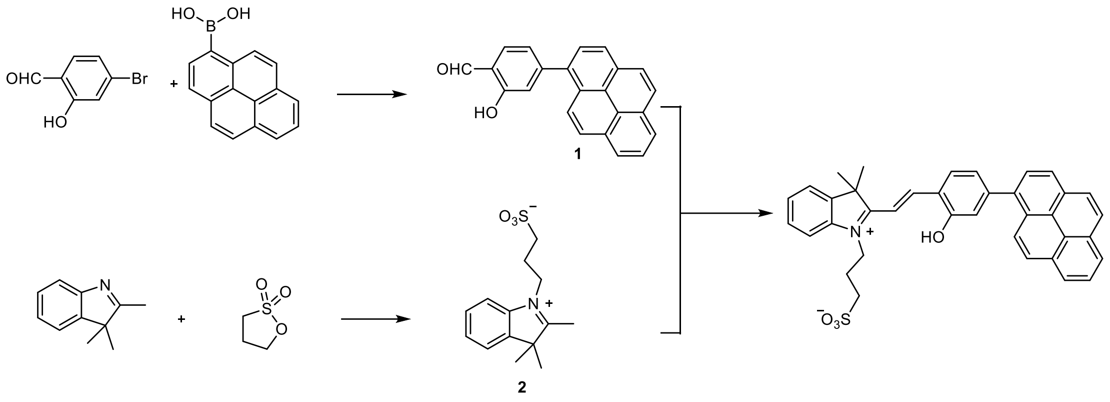

Supplementary Fig.1. The synthetic route of PMC.

DFT calculations: All the DFT calculations were performed by the Gaussian 16 package[2]. The molecular orbital energies of PMC and PSP were performed with B3LYP functional with 6-31+G basis set.[3] For molecular dynamics, geometry optimization of all structures was performed using the M06-2X functional[4], with the 6-31+G(d,p) basis set[5-7]. Frequency calculations were performed to obtain the Gibbs free energy correction at 298.15 K. Single point calculations were performed with the 6-311+G(d,p) basis set[8]. The SMD implicit solvation model[9] was used for all calculations with DMSO as the solvent. The molecular geometry and isosurface were plotted with CYLView[10].

^{1}H-NMR test of PSP: PMC (10 mg) is dissolved in 500 ml $CHCl_3$ and irradiation by the 500 nm light (~15 mW cm^{-2}) about 1 hour (according to the UV-Vis spectra results). Then the solution was concentrated quickly in room temperature (20-25 ºC). Obtained solid was dissolved in DMSO-d_6 for ^{1}H-NMR test. The ^{1}H-NMR spectra of PSP are shown in Fig. 2**b** and Supplementary Fig.30.

B. Supplementary Notes 1. Photophysical properties of PMC and PSP.

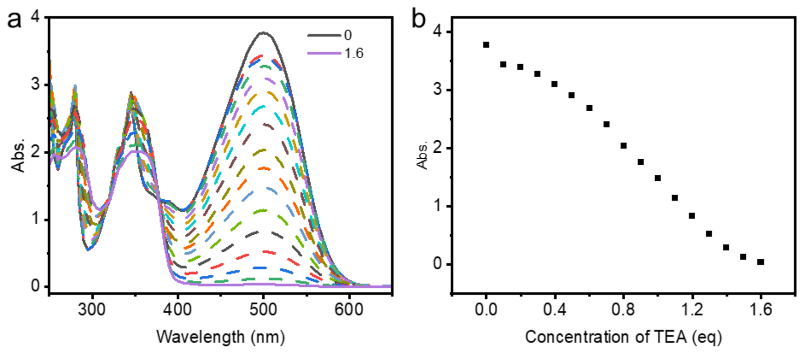

Supplementary Fig.2. The alkali titration of PMC in $CHCl_3$. UV-Vis spectra (**a**) and variation of absorbance (**b**) at 500 nm of PMC by adding triethylamine from 0 eq to 1.6 eq.

The absorbance at 500 nm is close to 0, indicating that PMC can completely transform into PSP upon adding excess triethylamine as an organic base. The generated PSP shows negligible contribution to the absorbance at 500 nm, so that we can determine [PMC] accurately according to the absorbance at 500 nm.

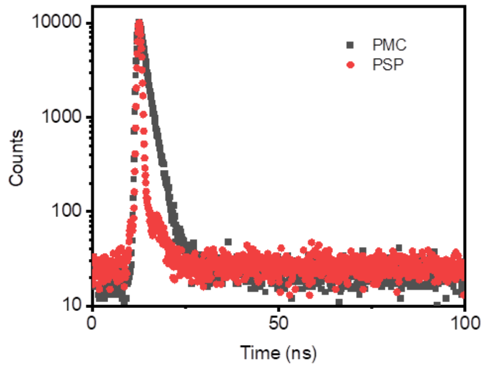

Supplementary Fig.3. Fluorescence lifetime of photoswitch in PMC state and PSP state with the concentration at 0.1 mM.

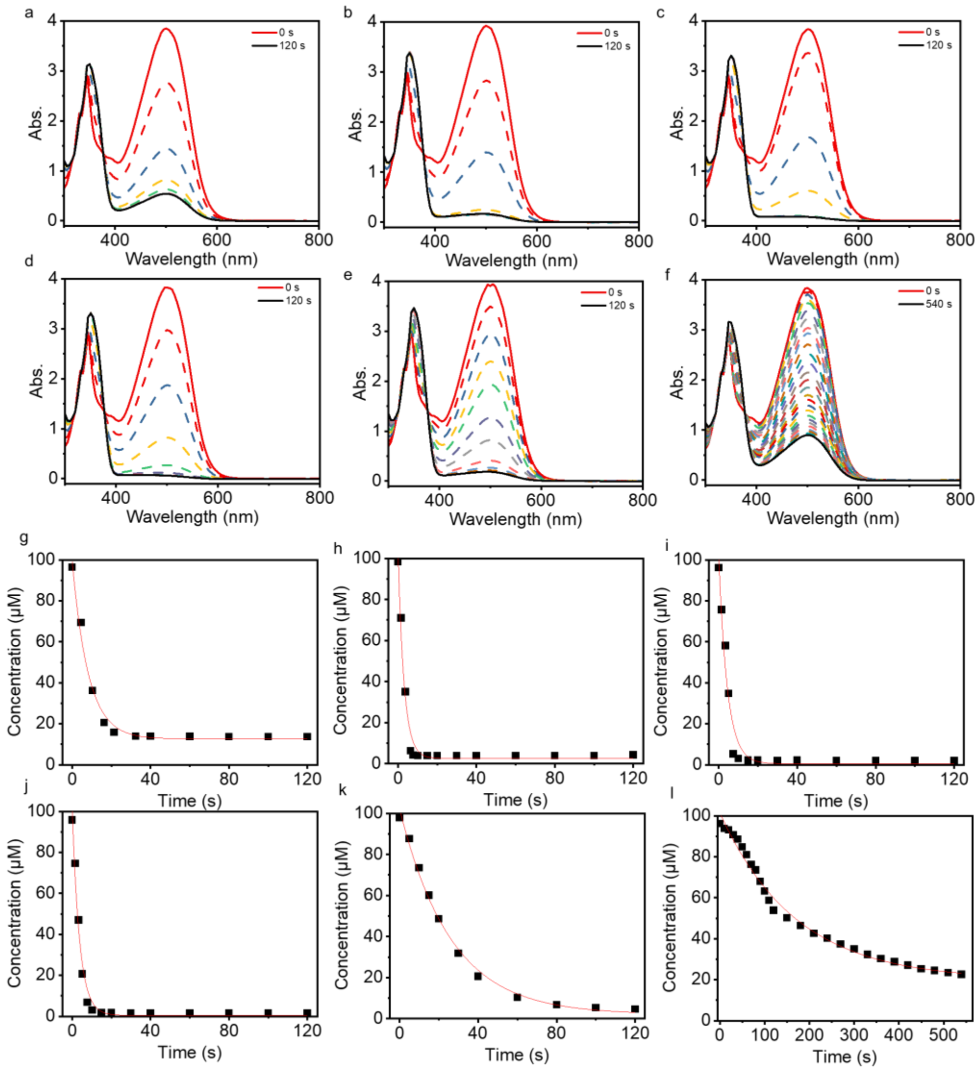

Supplementary Fig.4. The investigation of the photoisomerization processes of PMC with varying light wavelengths. UV-Vis spectra and nonlinear fitted plot of photoisomerization processes of PMC state (red line) and obtained PSP state (black line) upon irradiation with different light wavelength at 420 nm (**a, g**), 475 nm (**b, h**), 500 nm (**c, i**), 550 nm (**d, j**), 600 nm (**e, k**) and 650 nm (**f, l**) in $CHCl_3$.

Supplementary Table 1. The absorbance and concentration of photoswitch in chloroform before irradiation and after irradiation, absorbed photon flux, photoisomerization extent and quantum yield on photoisomerization processes with different light wavelength.

**Irradiation wavelength**	**Absorbed photon flux (μmol/m²/s)** [a]	**$[PMC]_0$ (μM)**	**[PMC] after irradiation (μM)**	**Initial photoreaction rate (μM/s)**	**Photoisomerization extent (%)**	**Quantum yield** [b]
**White light**	1240	97.95	4.86	29.39	95	0.24
**420 nm**	380	96.43	13.61	11.57	86	0.30
**475 nm**	500	98.36	3.88	32.46	96	0.64
**500 nm**	530	96.08	2.08	21.14	97	0.40
**550 nm**	824	95.75	1.57	25.85	98	0.31
**600 nm**	897	97.86	4.66	3.71	95	0.041
**650 nm**	982	96.10	22.51	0.57	77	0.006

Note:

a, the Photon flux is measured by a quantum par meter (AZ Instrument crop., AZ8583) before and after putting the quartz cell with solution as soon as possible.

b, the quantum yield is calculated from initial rate divided by absorbed photon flux[11], the light contact surface is 3×10^{-4} m² and the volume of solution is 3 mL.

C. Supplementary Notes 2. Investigation of ICT and self-assembly behavior of PMC.

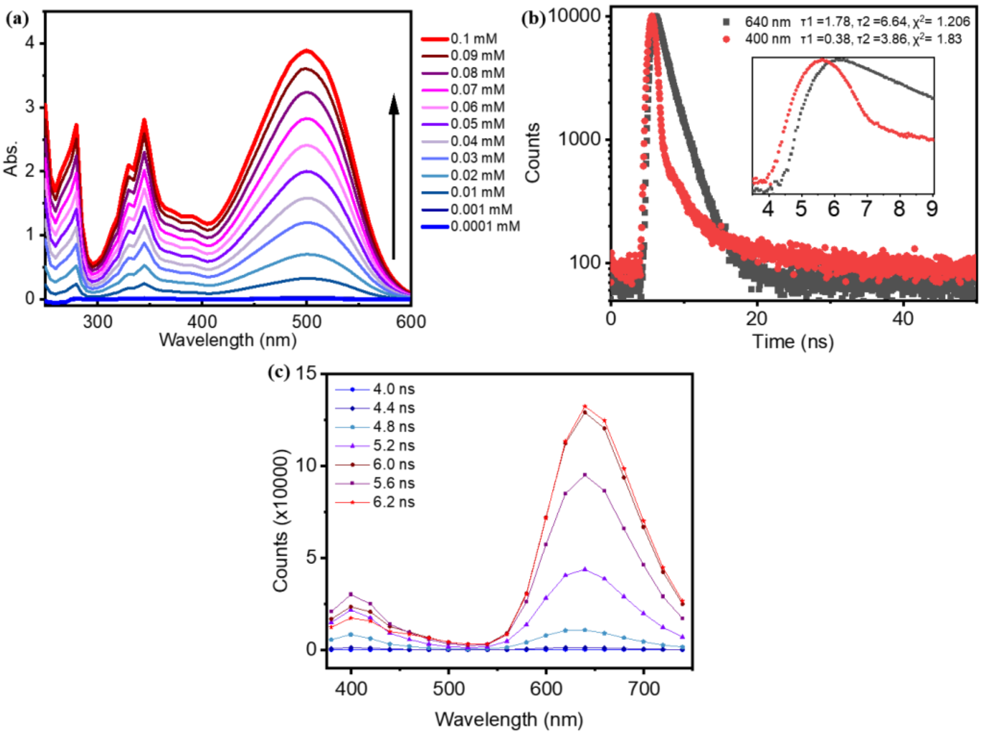

Supplementary Fig.5. The investigation of the UV-Vis spectra, life decay and time-resolved fluorescence spectra of the photoswitch in $CHCl_3$. (**a**) UV-Vis spectra of photoswitch in PMC state with varying concentration from 0.1 mM, 0.09 mM, 0.08 mM, 0.07 mM, 0.06 mM, 0.05 mM, 0.04 mM, 0.03 mM, 0.02 mM, 0.01 mM, 0.001 mM, and 0.0001 mM in $CHCl_3$. (**b**) The lifetimes for the emission at 640 nm and 400 nm of photoswitch in PMC state with the concentration at 0.03 mM in $CHCl_3$. Insert: the partial spectra. (**c**) The time-resolved fluorescence spectra of photoswitch in PMC state with the concentration at 0.03 mM. The excitation wavelength of (**b**) and (**c**) is 365 nm.

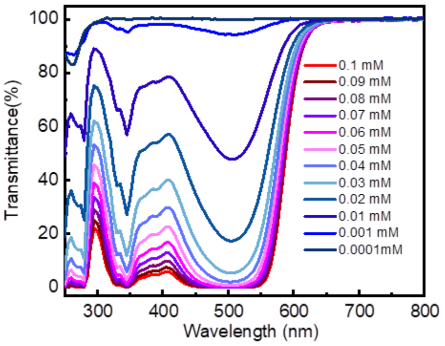

Supplementary Fig.6. Transmittance of photoswitch in PMC state with varying concentration from 0.1 mM, 0.09 mM, 0.08 mM, 0.07 mM, 0.06 mM, 0.05 mM, 0.04 mM, 0.03 mM, 0.02 mM, 0.01 mM, 0.001 mM, and 0.0001 mM in $CHCl_3$.

Supplementary Table 2. The element content of scan dot from SEM-EDS in $CHCl_3$.

	Dot 1		Dot 2	
Element	Wt%	At%	Wt%	At%
C	70.48	77.00	14.93	28.57
O	23.92	19.62	2.93	4.21
S	3.53	1.45	0.00	0.00
N	2.07	1.94	0.00	0.00
Si	0.00	0.00	82.14	67.22
Total	100.00	100.00	100.00	100.00

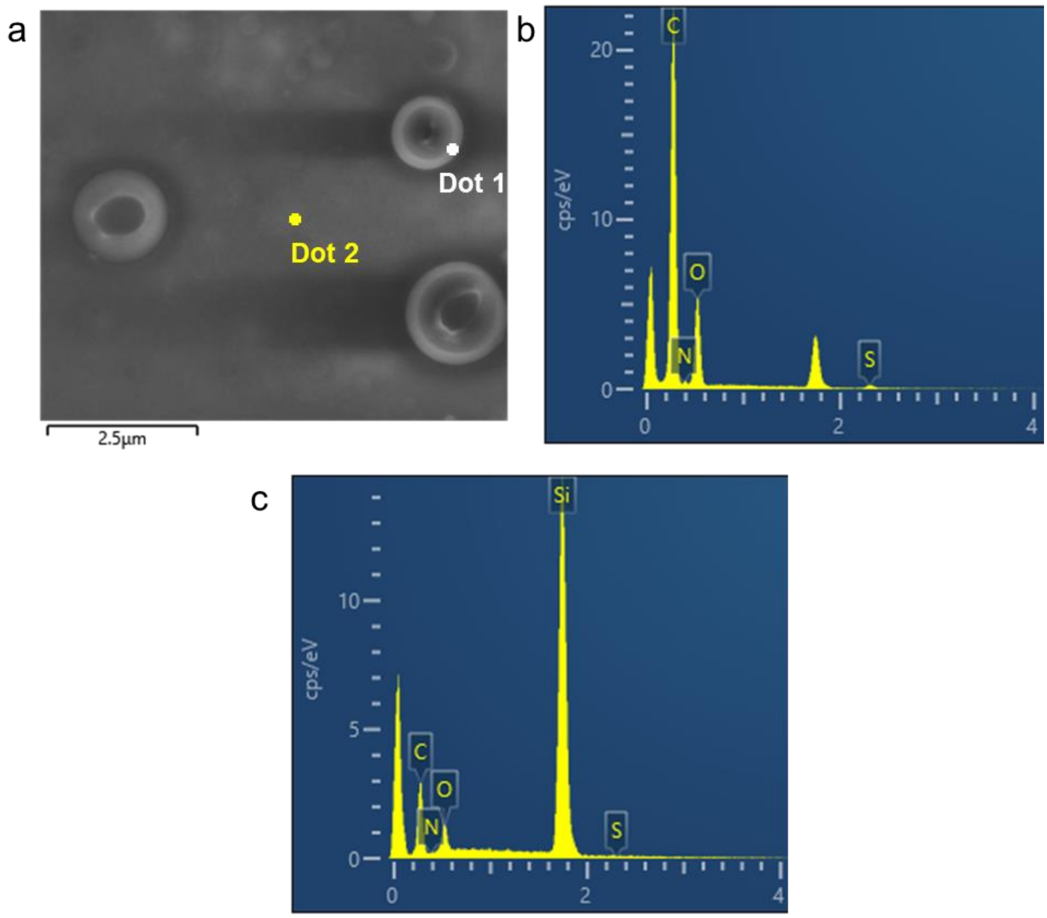

Supplementary Fig.7. The SEM-EDS analysis of PMC self-assemblies. (**a**) The dot scan of SEM-EDS of self-assemblies of PMC. The element analysis of dot 1(**b**) and dot 2 (**c**).

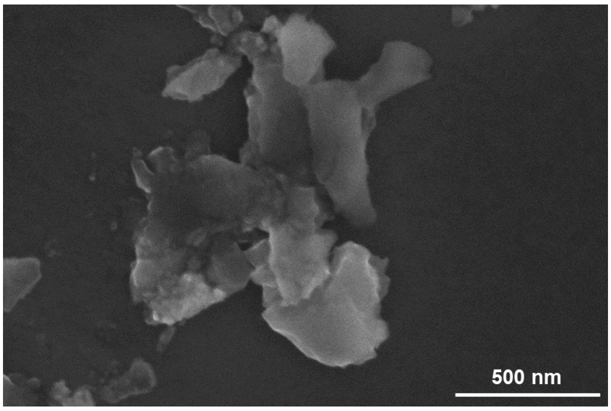

Supplementary Fig.8. The SEM image of the dissociation state (amorphous state) of PSP in $CHCl_3$.

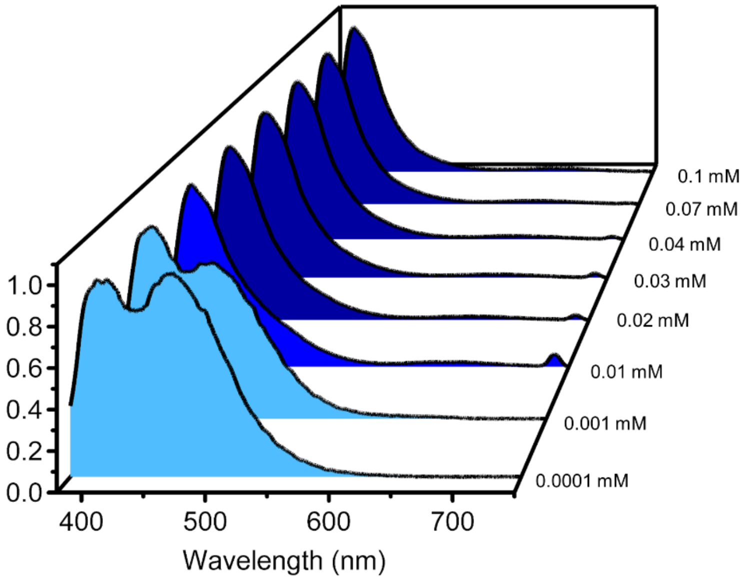

Supplementary Fig.9. Normalized fluorescence waterfall plot of PSP with varying concentration from 0.1 mM, 0.07 mM, 0.04 mM, 0.03 mM, 0.02 mM, 0.01 mM, 0.001 mM, and 0.0001 mM in $CHCl_3$ (The excitation wavelength is 365 nm).

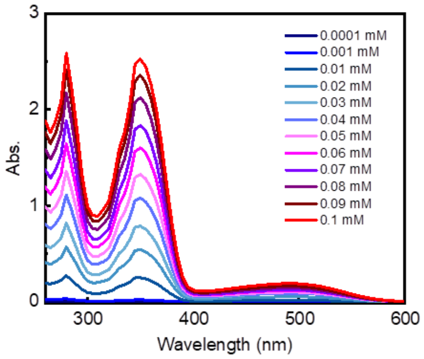

Supplementary Fig.10. UV-Vis absorption of PSP with varying concentration from 0.1

mM, 0.09 mM, 0.08 mM, 0.07 mM, 0.06 mM, 0.05 mM, 0.04 mM, 0.03 mM, 0.02 mM, 0.01 mM, 0.001 mM, and 0.0001 mM in $CHCl_3$.

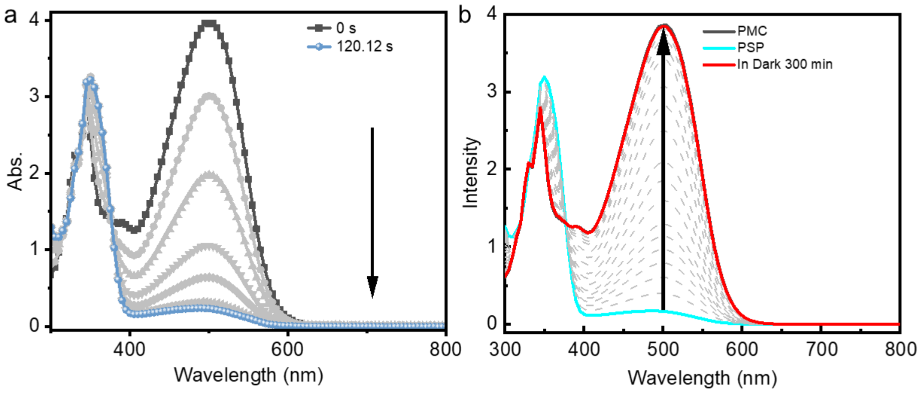

Supplementary Fig.11. Time-dependent UV-Vis absorption spectra of photoisomerization and thermal relaxation of the photoswitch in $CHCl_3$. (**a**) photoisomerization from PMC state to PSP state. (**b**) thermal relaxation from PSP state to PMC state.

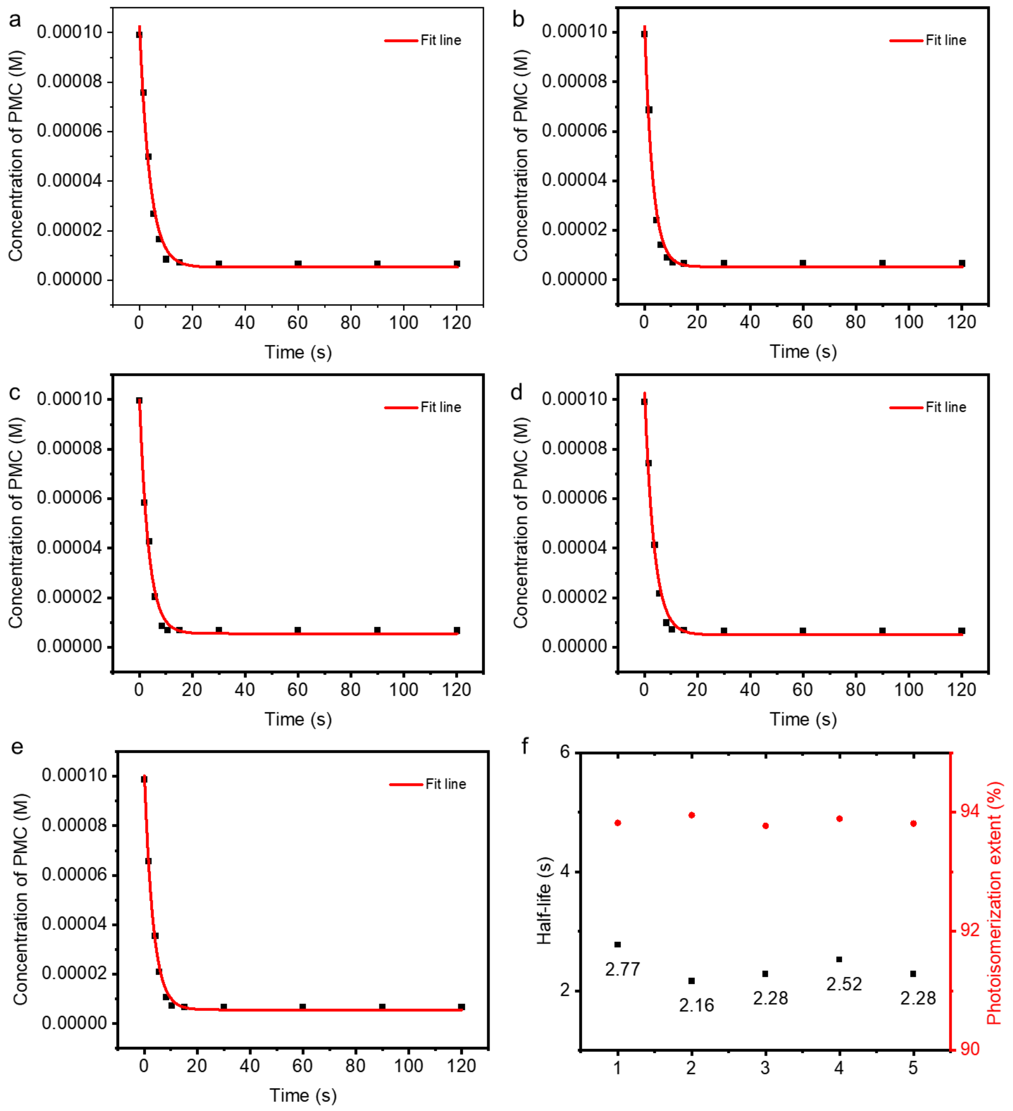

Supplementary Fig.12 The time-dependent concentration of PMC during photoisomerization process. (**a-e**) Nonlinear fitted plot of five photoisomerization processes and (**f**) the half-lives and photoisomerization extent of five light irradiation process.

The parameters of fit lines are shown in Supplementary Table 3. The photoisomerization processes of PMC in $CHCl_3$ are carried out via while light (the incident photon flux is 1545 ± 50 μmol/m^2/s).

Supplementary Table 3. The parameter of first-order kinetic equation applying on the five photoisomerization processes.

Equation	$[PMC] = [\mathrm{PMC_0}] \times e^{(-k_1 t)} + \mathrm{C}\ (1)$				
**C (μM)**	5.28 ± 1.64	5.24 ± 1.64	5.57 ± 1.38	5.05 ± 1.69	5.62 ± 0.96
**PMC_0 (μM)**	97.30 ± 3.56	97.16 ± 3.92	94.35 ± 3.24	97.60 ± 3.80	94.61 ± 2.22
**k_1 (1/s)**	0.26 ± 0.02	0.33 ± 0.03	0.30 ± 0.02	0.28 ± 0.02	0.30± 0.02
**R^2**	0.9899	0.98754	0.99104	0.98852	0.99577

Note: **t** is irradiation time, **C** is correction coefficient, and **k_1** is rate constant of photoisomerization process.

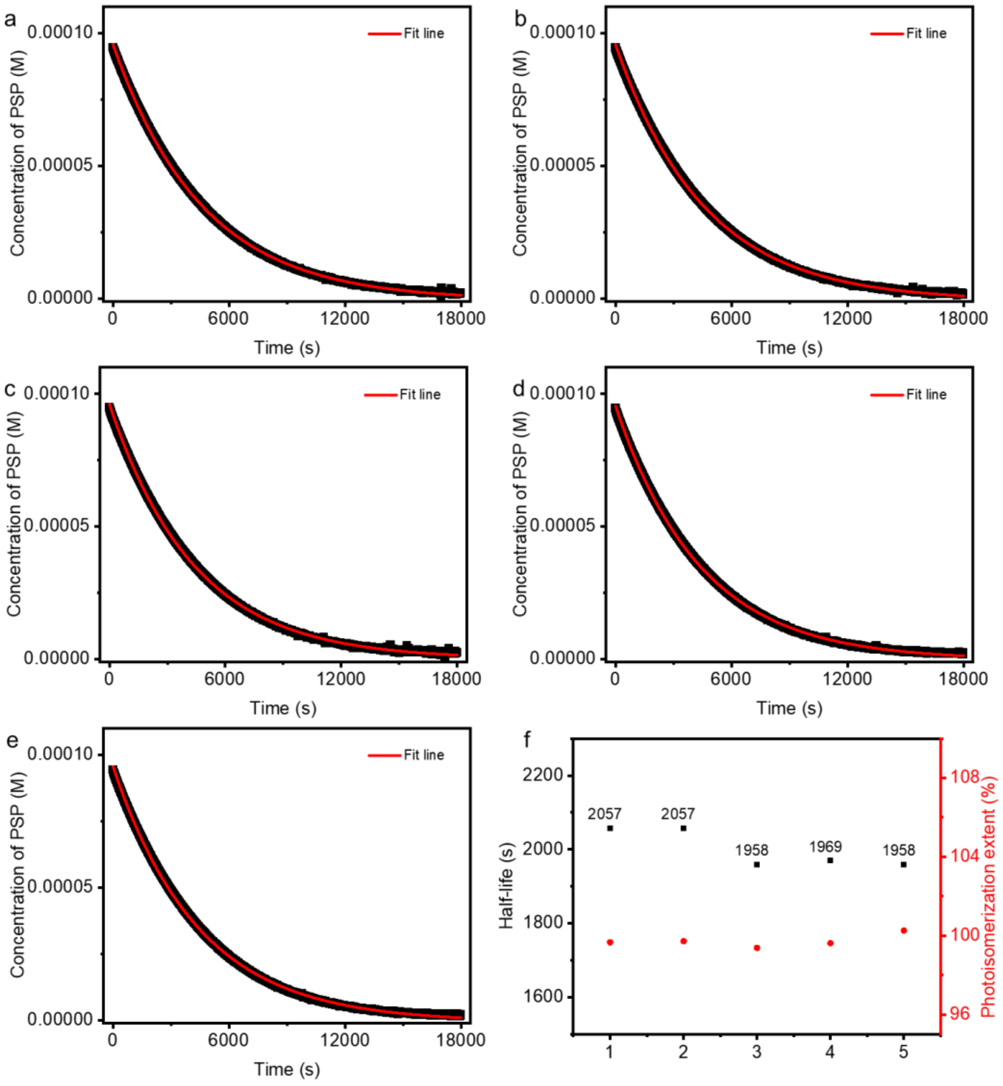

Supplementary Fig.13. The time-dependent concentration of PSP during thermal relaxation process. (**a-e**) Nonlinear fitted plots of five thermal relaxation processes and (**f**) the half-lives and photoisomerization extent of five thermal relaxation processes.

The parameters of fit lines are shown in Supplementary Table 4.

Supplementary Table 4. The parameters of first-order kinetic equation applying on thermal relaxation processes.

**Equation**	$[PSP] = [PSP_0] \times e^{(-k_2 t)} + C$ (2)				
**C (μM)**	–0.7 ± 0.02	–0.9 ± 0.03	–0.7 ± 0.03	–0.04 ± 0.03	–0.8 ± 0.03
**PSP_0 (μM)**	96.35 ± 0.04	96.48 ± 0.05	96.16± 0.05	95.75 ± 0.05	96.18 ± 0.05
**k_2 (1/s)**	216.04 ± 0.23	218.17 ± 0.27	228.04 ± 0.31	227.55 ± 0.27	225.68 ± 0.30
**R^2**	0.99971	0.99961	0.99951	0.99961	0.99954

Note: **t** is thermal relaxation time, **C** is correction coefficient, and **k_2** is rate constant of thermal relaxation process.

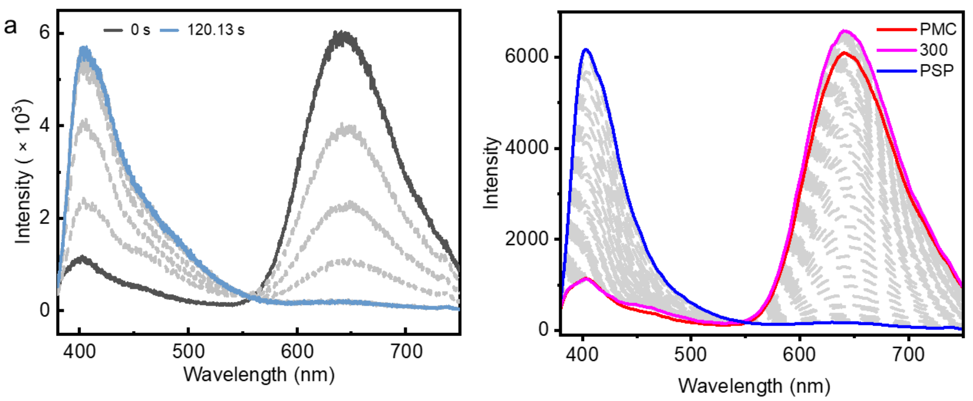

Supplementary Fig.14. The fluorescence spectra of time-dependent photoisomerization and thermal relaxation processes of the photoswitch in $CHCl_3$. (**a**) Photoisomerization from PMC state to PSP state. (**b**) Thermal relaxation from PSP state to PMC state.

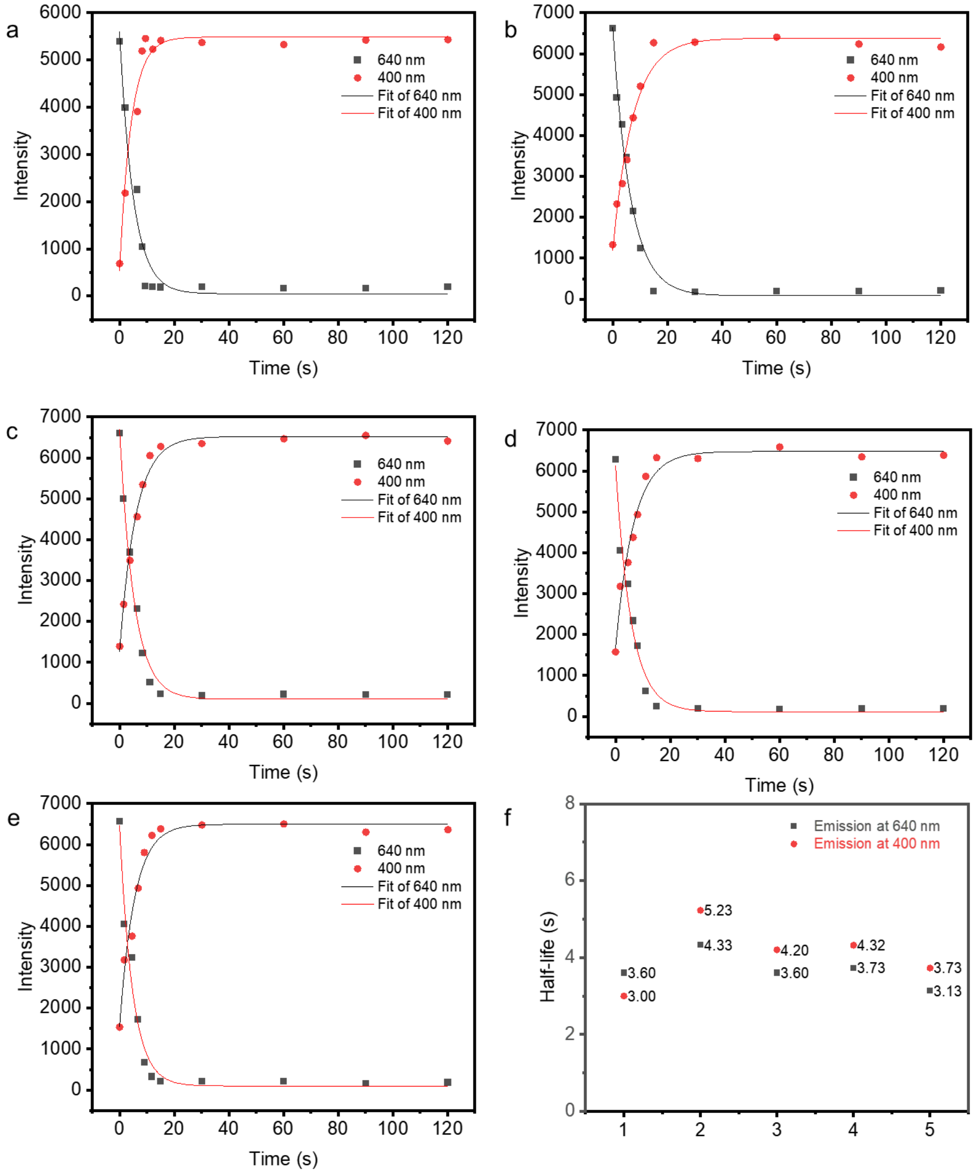

Supplementary Fig.15. The fluorescence variation of photoisomerization process of the photoswitch in $CHCl_3$. (a-e) Nonlinear fitted plots of five photoisomerization processes and **(f)** the half-lives of five photoisomerization processes with emissions at 640 nm and 400 nm. We fitted the fluorescence intensity variation by first-order kinetic equation according to UV-Vis spectra. The parameters of fit lines are shown in Supplementary Table 5.

Supplementary Table 5. The parameters of first-order kinetic equation for photoisomerization processes based on time-dependent fluorescence intensities.

Equation		$I = I_0 \times e^{(-kt)} + C\ (3)$				
Emission at 640 nm	**C**	55.58 ± 136.81	82.81 ± 159.46	100.30 ± 122.47	115.13 ± 150.92	97.66 ± 161.40
	I_0	5600.33 ± 305.27	6566.14 ± 296.95	6596.86 ± 241.37	6007.40 ± 297.88	6368.52 ± 337.62
	**k** (1/s)	0.24 ± 0.03	0.15 ± 0.02	0.19 ± 0.02	0.18 ± 0.02	0.21 ± 0.03
	R^2	0.97771	0.98469	0.98975	0.98136	0.97868
Emission at 400 nm	**C**	5476.33 ± 135.12	6375.39 ± 154.40	6520.11 ± 94.82	6477.14 ± 136.80	6496.67 ± 138.35
	I_0	-5000.12 ± 321.04	-5189.72± 276.52	-5257.56 ± 180.77	-4796.18 ± 259.70	-4949.60± 276.39
	**k** (1/s)	0.29 ± 0.04	0.14 ± 0.02	0.17 ± 0.01	0.15 ± 0.02	0.18 ± 0.02
	R^2	0.96928	0.97884	0.99093	0.9779	0.97637

Note: **t** is irradiation time, **C** is correction coefficient, and **k** are rate constants of fluorescence emission variation.

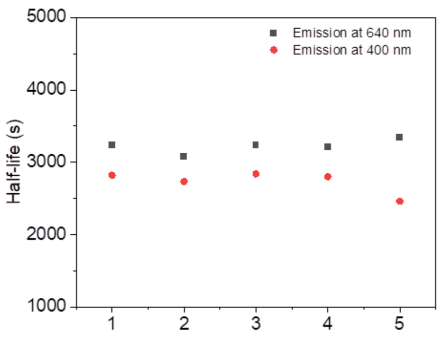

Supplementary Fig.16. The half-lives of five thermal relaxation processes with emissions at 640 nm and 400 nm.

D. Supplementary Notes 3. Solvent effect of photochromic fluorescence of PMC.

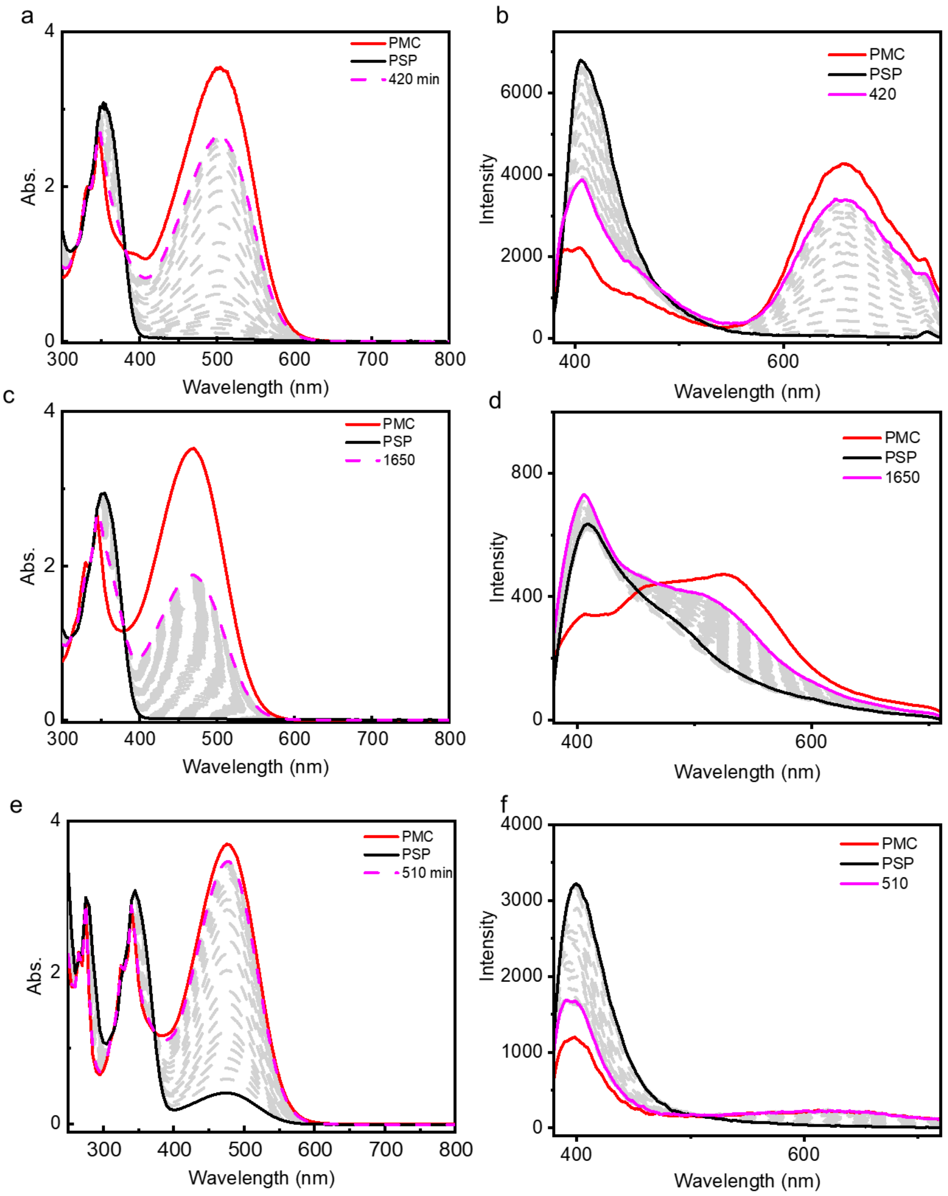

Supplementary Fig.17. The investigation of the thermal relaxation process of the photoswitch in DCB, DMSO, and EtOH. (a, b) The UV-Vis and fluorescence spectra of photoisomerization and thermal relaxation processes in DCB. (**c, d**) The UV-Vis and fluorescence spectra of photoisomerization and thermal relaxation processes in DMSO. (**e, f**) The UV-Vis and fluorescence spectra of photoisomerization and thermal relaxation processes in EtOH.

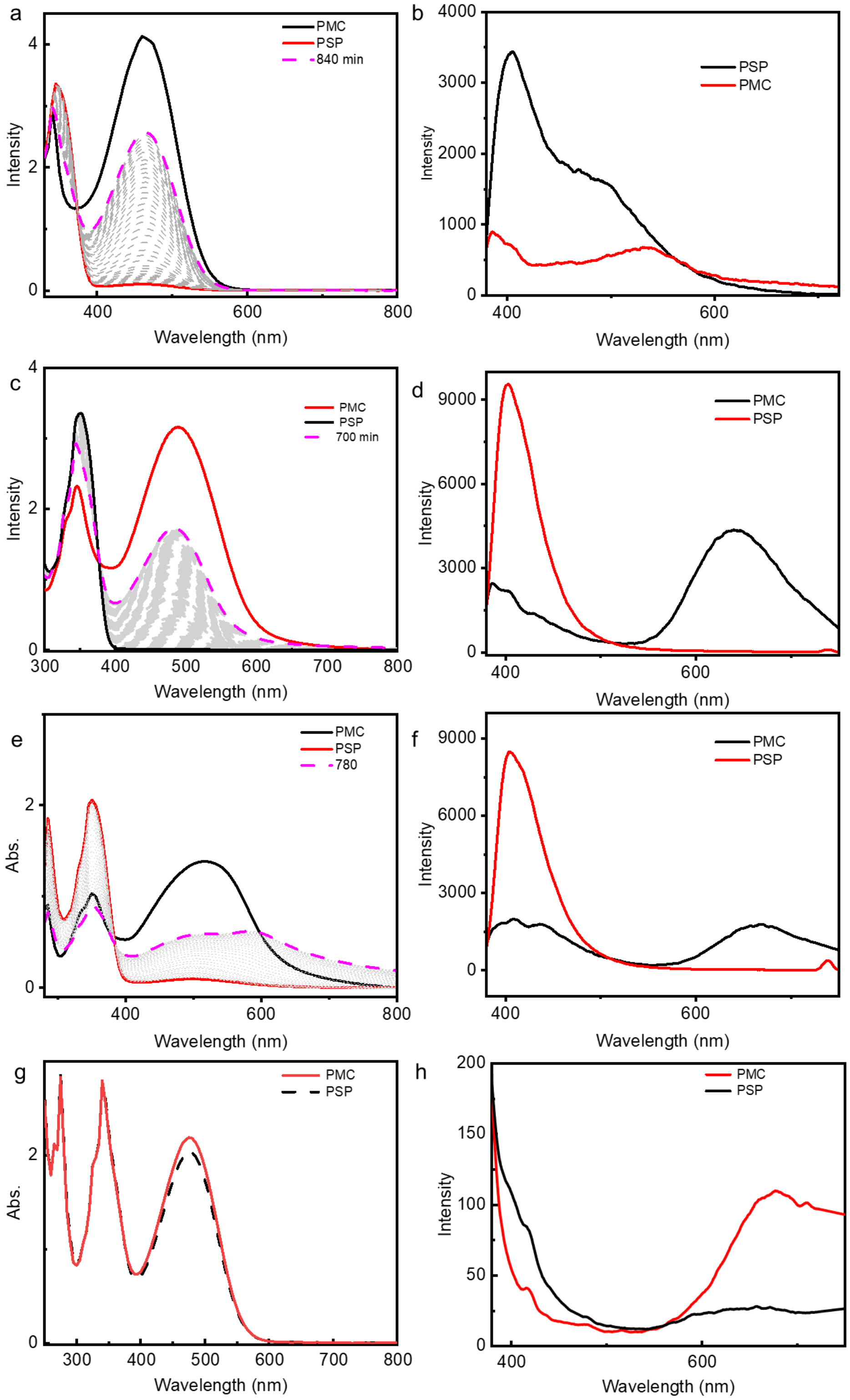

Supplementary Fig.18. The investigation of the thermal relaxation processes of photoswitch in DMK, 1,4-dioxane, toluene, and H_2O. (**a, b**) The UV-Vis of photoisomerization and thermal relaxation processes and fluorescence spectra for photoswitch in DMK. (**c, d**) The UV-Vis of photoisomerization and thermal relaxation processes and fluorescence spectra for photoswitch in 1,4-dioxane, (**e, f**) The UV-Vis

of photoisomerization and thermal relaxation processes and fluorescence spectra for photoswitch in toluene. (**g, h**) The UV-Vis and fluorescence spectra of photoswitch in H_2O.

Supplementary Table 6. Summary for maximum absorption and photoisomerization extent of the photoswitch in different solvents based on UV-Vis spectra.

Solvent	Maximum absorption wavelength (nm)	Maximum absorbance before irradiation	Maximum absorbance after irradiation	Photoisomerization extent of light irradiation (%)[a]	Maximum absorbance after thermal relaxation	Photoisomerization extent of thermal relaxation (%)[b]
**DMK**	465	4.10	0.10	98	2.57	63
**DMSO**	470	3.52	0.02	99	1.88	53
**1,4-Dioxane**	490	3.15	0.02	99	1.71	54
**o-DCB**	505	3.54	0.04	99	2.66	75
**ETOH**	475	3.69	0.41	89	3.46	93
**Toluene**	515	1.37	0.09	93	0.59	43

a, b According to the experimental results, PSP shows negligible contribution to the certain absorbance. Therefore, we consider the absorbance decreasing efficiency as the photoisomerization extent.

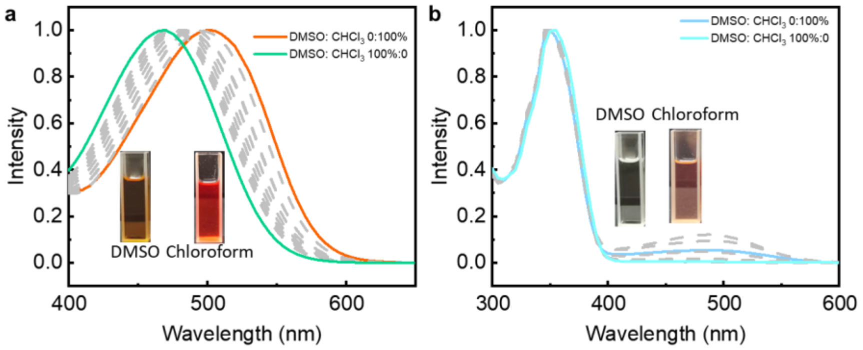

Supplementary Fig.19. UV-Vis spectra of photoswitch in DMSO/$CHCl_3$ mixed solvents. The UV-Vis spectra of photoswitch in PMC state (**a**) and PSP state (**b**) in DMSO/$CHCl_3$ mixed solvents with DMSO fraction from 0 to100%.

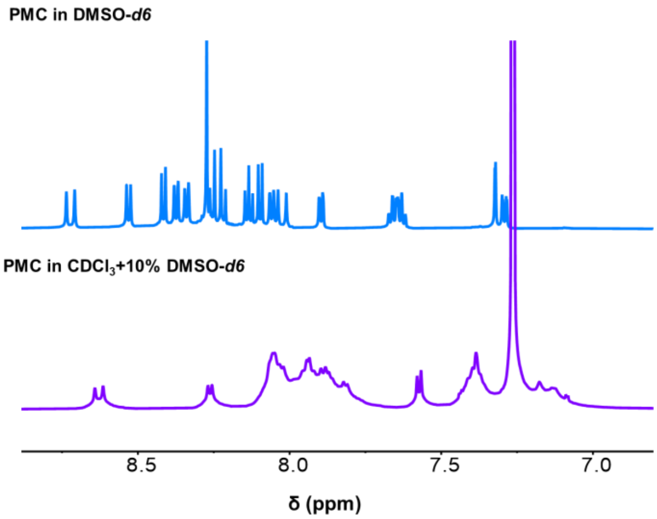

Supplementary Fig.20. The partial ^{1}H-NMR spectra of PMC in DMSO-d_6 and $CDCl_3$/DMSO-d_6 mixed solvents (V:V = 9:1).

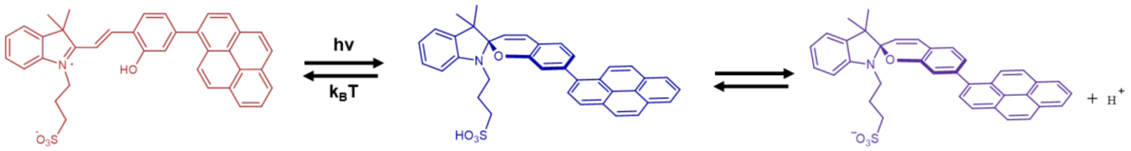

Supplementary Fig.21. Proposed chemical structures of photoisomerization and ionization process of PMC in ethanol.

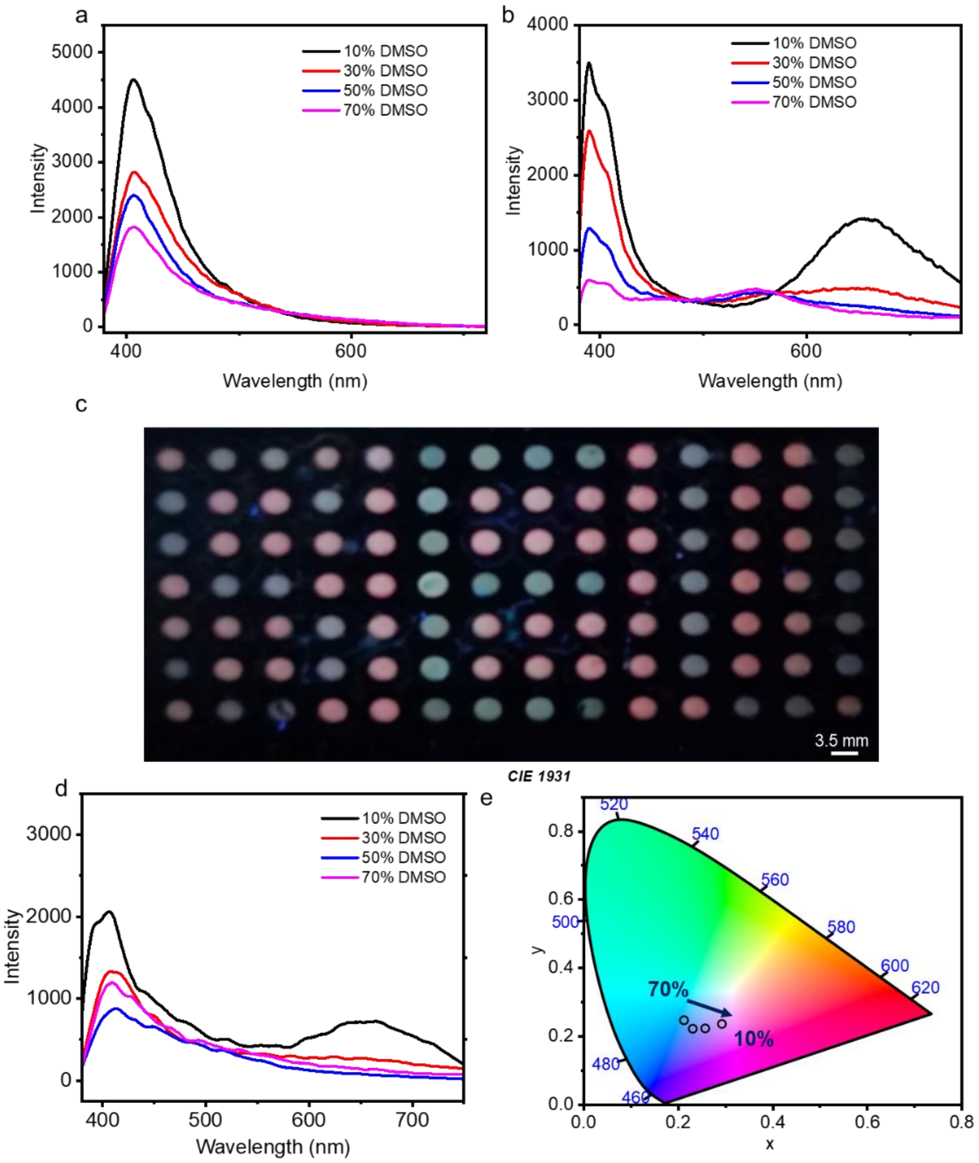

Supplementary Fig.22. Fluorescence variation of photoswitch in DMSO/DCB mixed solvents. Fluorescence spectra of (**a**) PSP and (**b**) PMC with different DMSO fraction in DMSO/DCB mixed solvents. (**c**) The digital images of anti-counterfeiting patterns after 12 hours. The scale bar is 3.5 mm. (**d**) fluorescence spectra and (**e**) CIE 1931 diagram of PSP with different DMSO fraction in DMSO/DCB mixed solvents after 12 hours.

E. Supplementary Notes 4. Photochromic fluorescence of PMC in PMMA film.

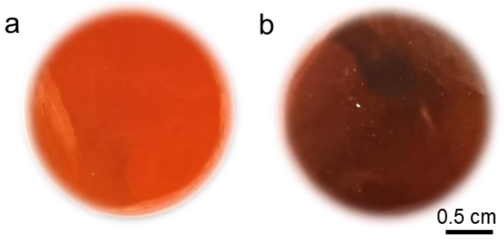

Supplementary Fig.23. Variation of photoswitch in PMMA film with light irradiation. Digital images of PMC/PMMA film (**a**) before and (**b**) after light irradiation. The scale bar is 0.5 cm.

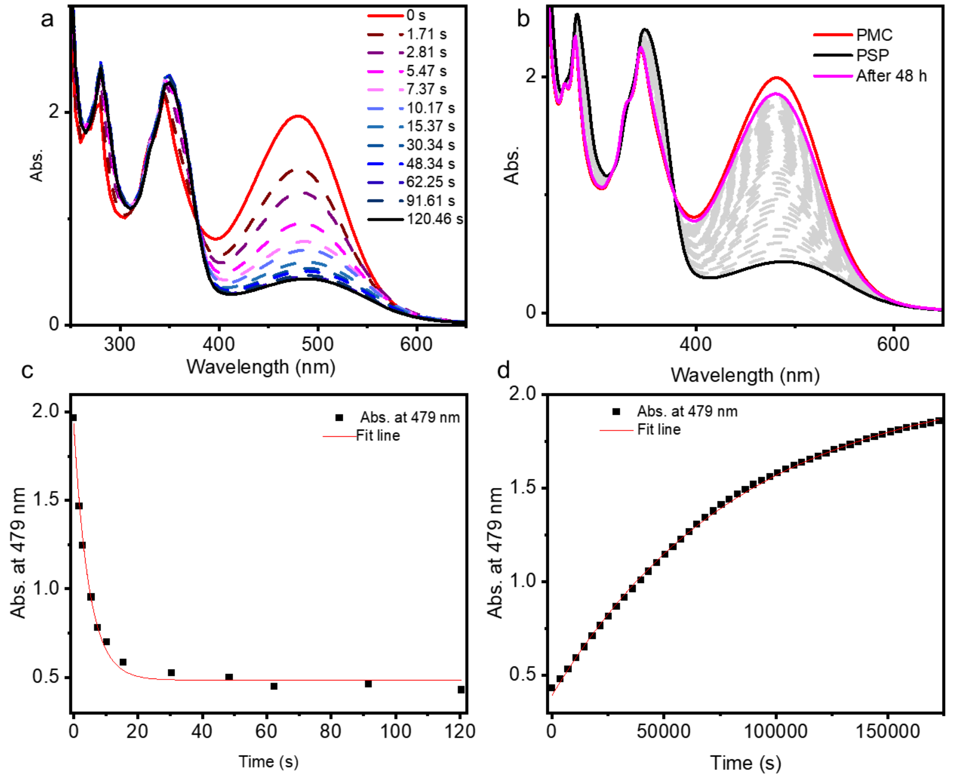

Supplementary Fig.24. The investigation of photoswitch in PMMA film. UV-Vis spectra of photoswitch in PMMA film during (**a**) photo-isomerization and (**b**) thermal relaxation process, and variation of absorbance of 479 nm during (**c**) photoisomerization and (**d**) thermal relaxation process. The fitting equation is $Abs. = 1.45 \times e^{(-0.21t)} + 0.48$ (4) ($R^2 = 0.995$) of (**c**) and $Abs. = -1.69 \times e^{(-1.17 \times 10^{-5} t)} + 2.08$ (5) (R^2 = 0.999), respectively, applying the first-order kinetic equation. The photoisomerization processes of PMC in PMMA film is carried out via while light with initial absorbed photon flux as 945 μmol/m^2/s.

F. Supplementary Figures

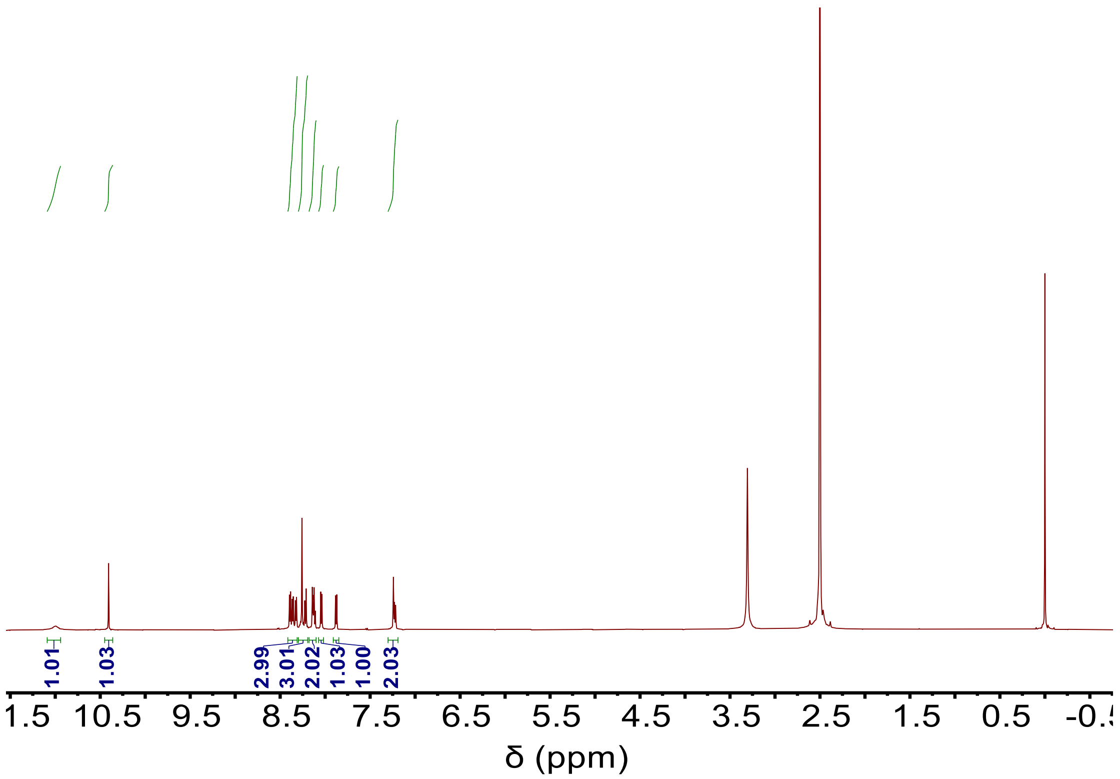

Supplementary Fig.25. The ^{1}H-NMR of 1 in DMSO-d_6 (600 MHz, 298 K).

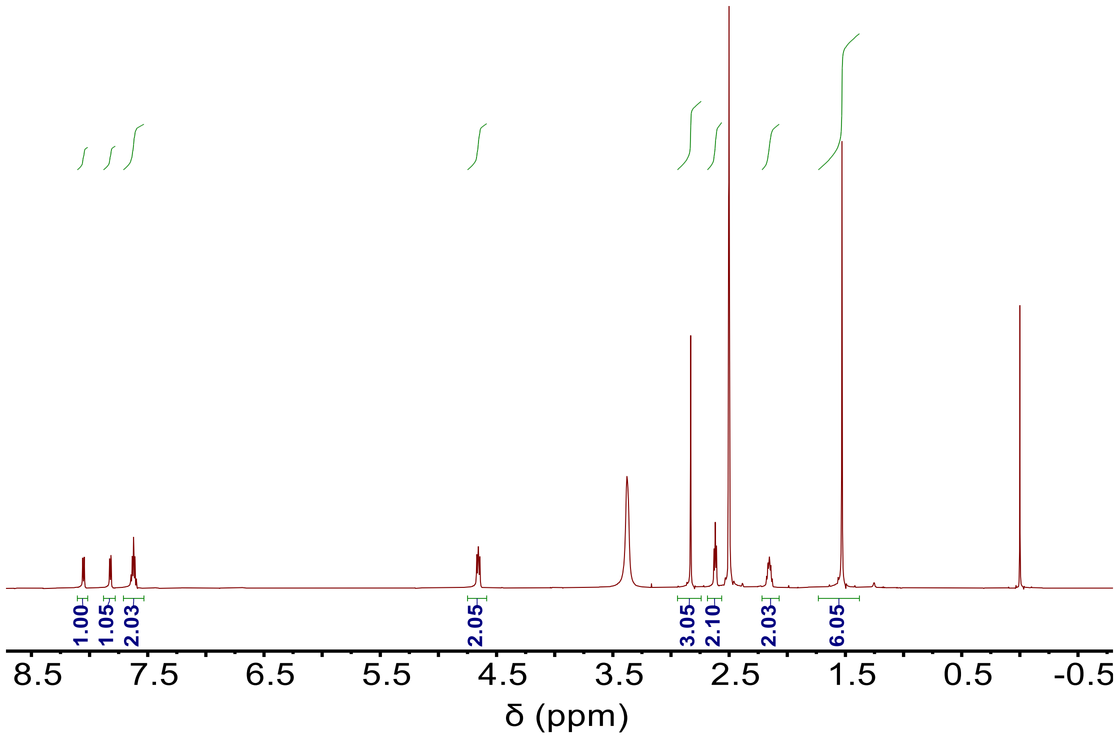

Supplementary Fig.26. The ^{1}H-NMR of 2 in DMSO-d_6 (600 MHz, 298 K).

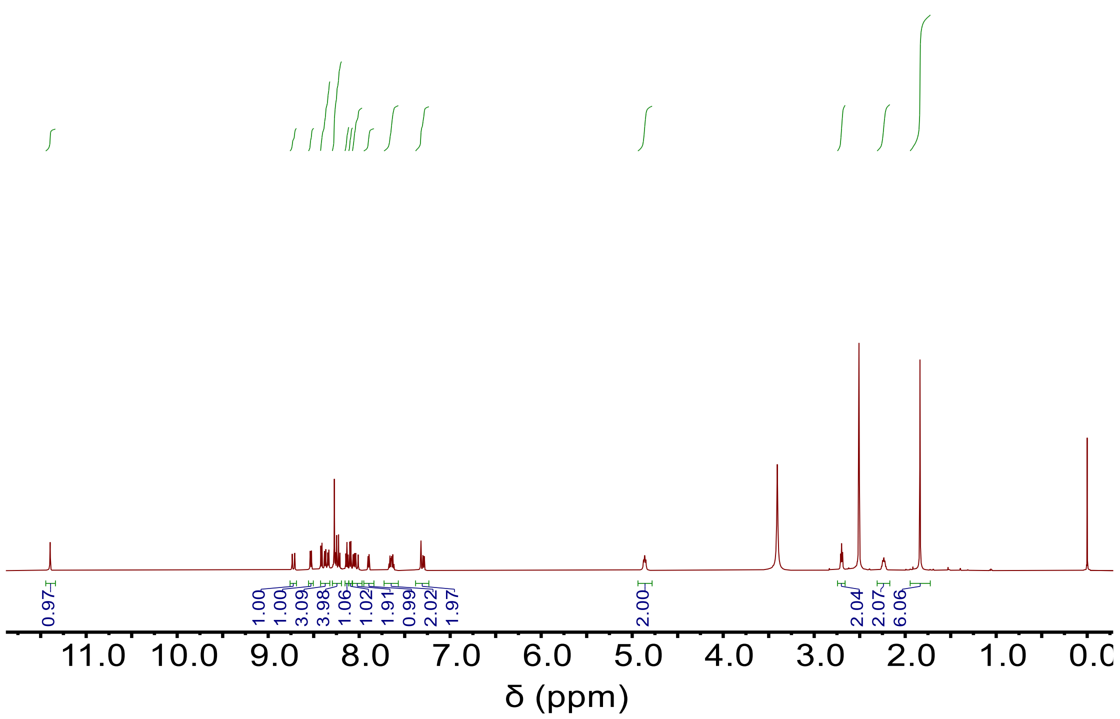

Supplementary Fig.27. The ^{1}H-NMR of PMC in DMSO-d_6 (600 MHz, 298 K).

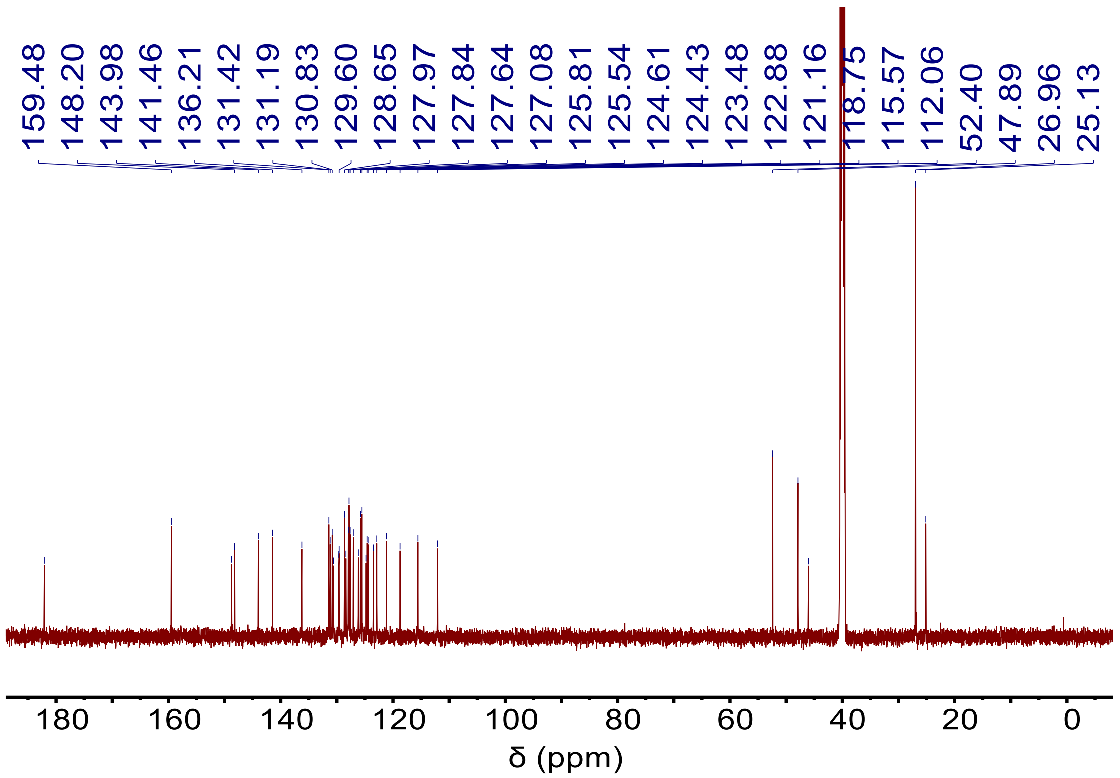

Supplementary Fig.28. The ^{13}C-NMR of PMC in DMSO-d_6 (151 MHz, 298 K).

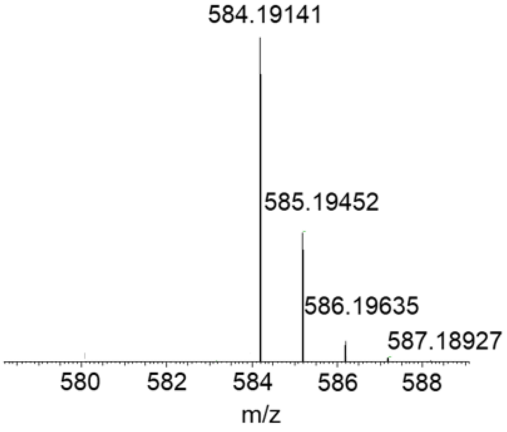

Supplementary Fig.29. The ESI-HRMS of PMC.

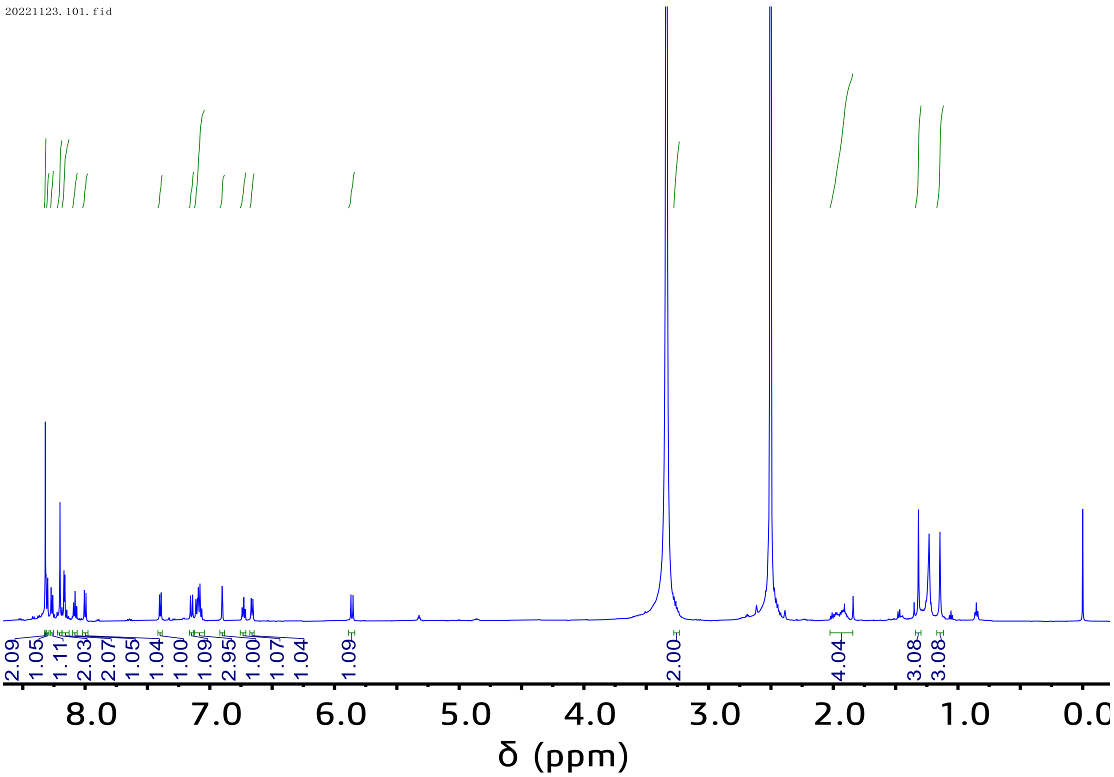

Supplementary Fig.30. The ^{1}H-NMR of (R)-3-(3',3'-dimethyl-7-(pyren-1-yl)spiro[chromene-2,2'-indolin]-1'-yl)propane-1-sulfonic acid (PSP) in DMSO-d_6 (600 MHz, 298 K).

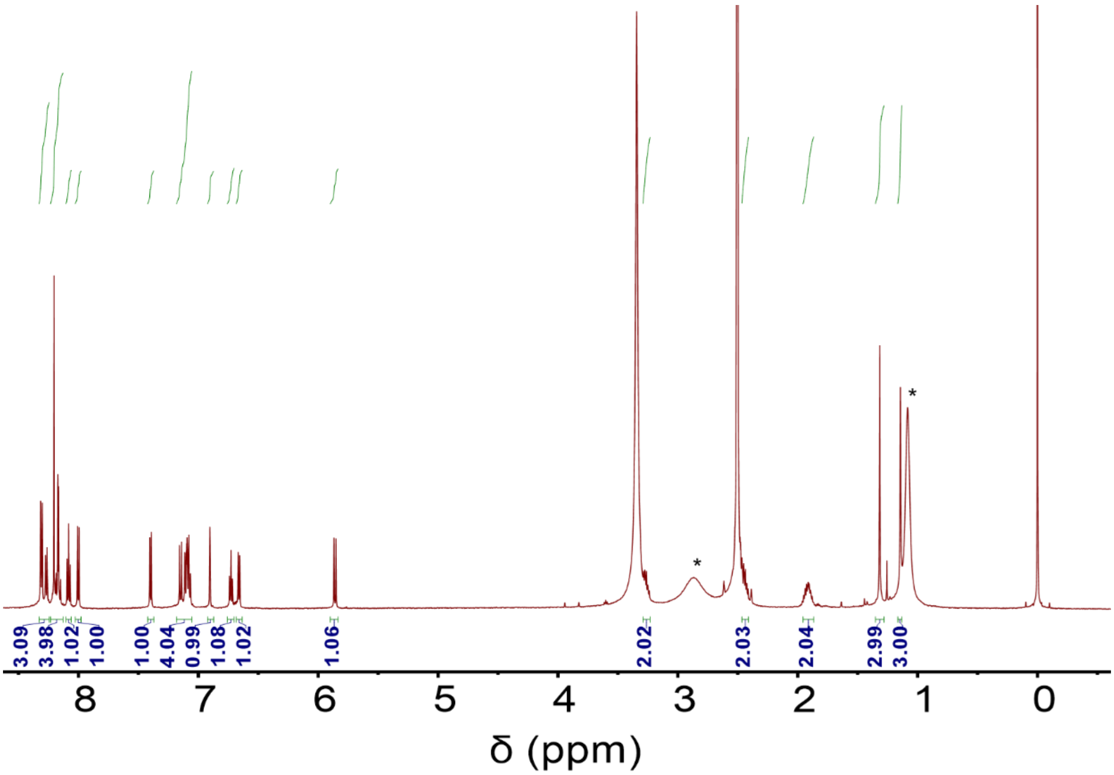

Supplementary Fig.31. The ^{1}H-NMR of (R)-3-(3',3'-dimethyl-7-(pyren-1-yl)spiro[chromene-2,2'-indolin]-1'-yl)propane-1-sulfonic acid (PSP) in DMSO-d_6 (600 MHz, 298 K) with triethylamine. The triethylamine is marked by asterisk.

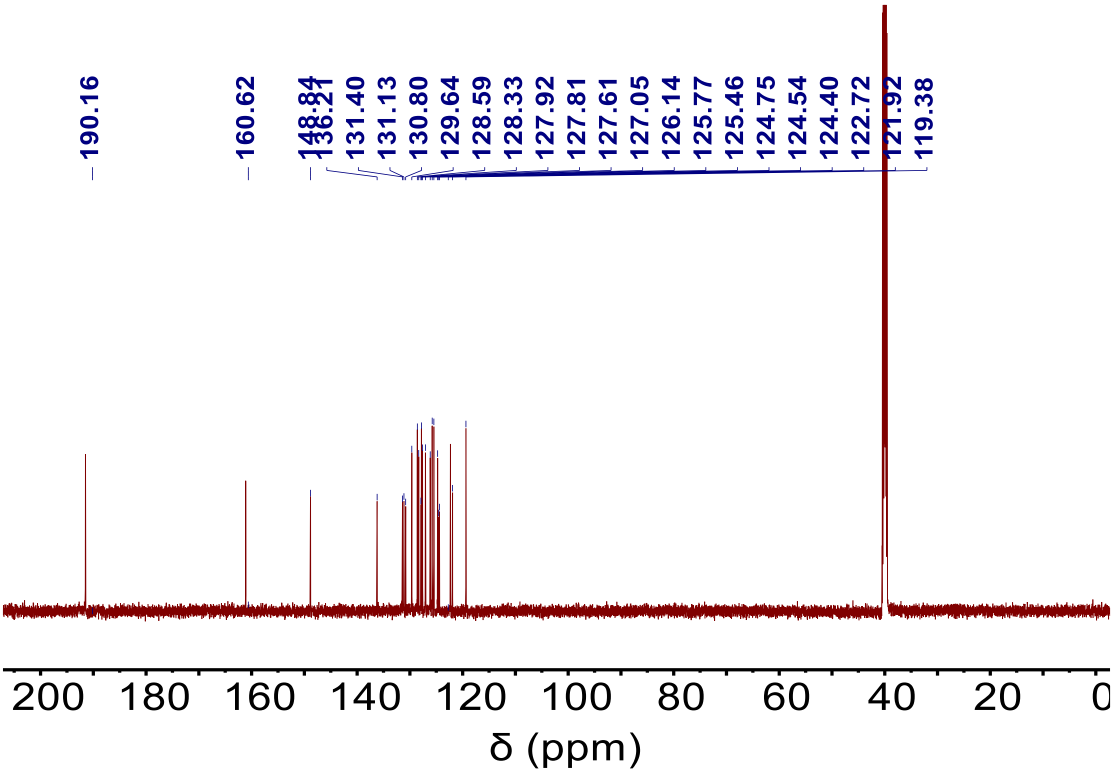

Supplementary Fig.32. The ^{13}C-NMR of 1 in DMSO-d_6 (151 MHz, 298 K).

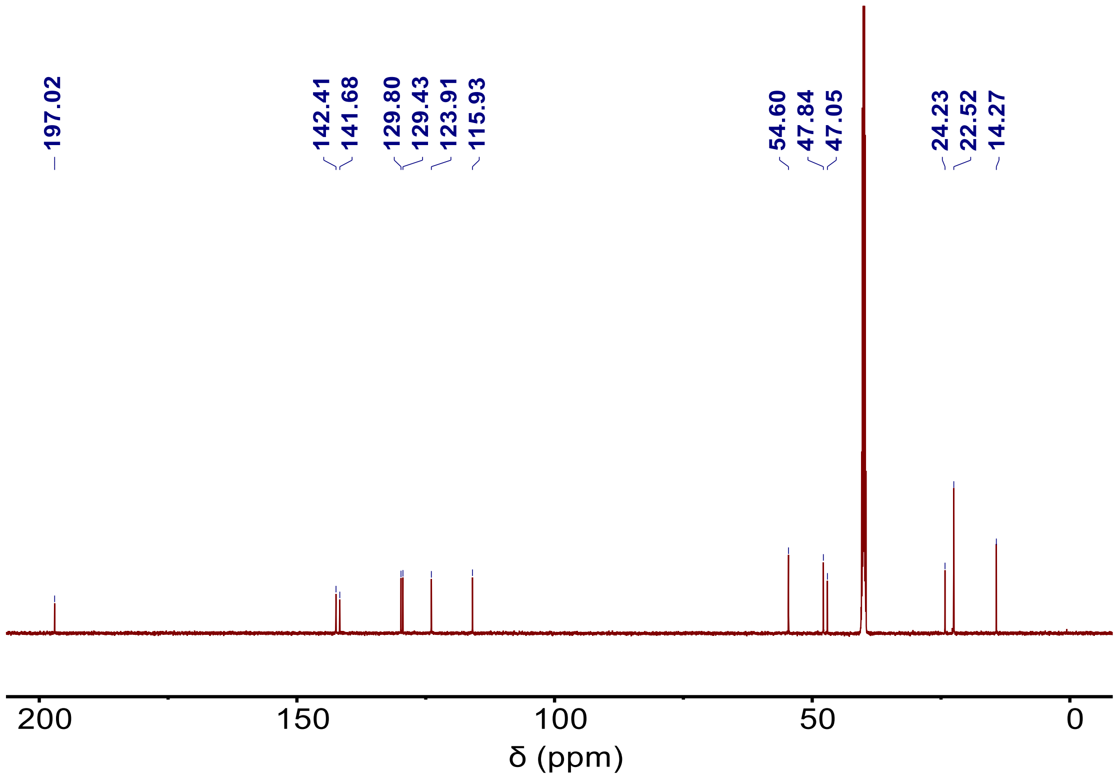

Supplementary Fig.33. The ^{13}C-NMR of 2 in DMSO-d_6 (151 MHz, 298 K).

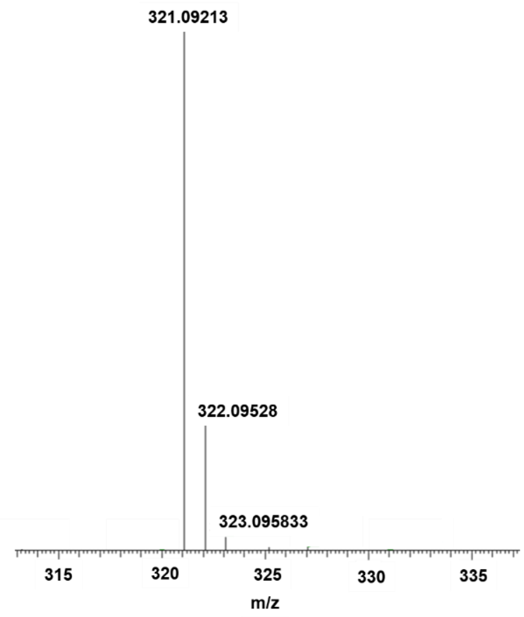

Supplementary Fig.34. The ESI-HRMS of 1.

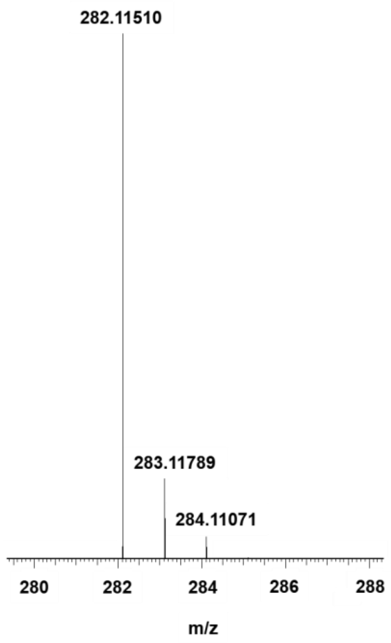

Supplementary Fig.35. The ESI-HRMS of 2.

Supplementary References:

1. Shi, Z.; Peng, P.; Strohecker, D. & Liao, Y. Long-lived photoacid based upon a photochromic reaction. *J. Am. Chem. Soc.* **133**, 14699–14703 (2011).
2. Frisch, M. J.; Trucks, G. W.; Schlegel, H. B.; Scuseria, G. E.; Robb, M. A.; Cheeseman, J. R.; Scalmani, G.; Barone, V.; Petersson, G. A.; Nakatsuji, H.; Li, X.; Caricato, M.; Marenich, A. V.; Bloino, J.; Janesko, B. G.; Gomperts, R.; Mennucci, B.; Hratchian, H. P.; Ortiz, J. V.; Izmaylov, A. F.; Sonnenberg, J. L.; Williams; Ding, F.; Lipparini, F.; Egidi, F.; Goings, J.; Peng, B.; Petrone, A.; Henderson, T.; Ranasinghe, D.; Zakrzewski, V. G.; Gao, J.; Rega, N.; Zheng, G.; Liang, W.; Hada, M.; Ehara, M.; Toyota, K.; Fukuda, R.; Hasegawa, J.; Ishida, M.; Nakajima, T.; Honda, Y.; Kitao, O.; Nakai, H.; Vreven, T.; Throssell, K.; Montgomery Jr., J. A.; Peralta, J. E.; Ogliaro, F.; Bearpark, M. J.; Heyd, J. J.; Brothers, E. N.; Kudin, K. N.; Staroverov, V. N.; Keith, T. A.; Kobayashi, R.; Normand, J.; Raghavachari, K.; Rendell, A. P.; Burant, J. C.; Iyengar, S. S.; Tomasi, J.; Cossi, M.; Millam, J. M.; Klene, M.; Adamo, C.; Cammi, R.; Ochterski, J. W.; Martin, R. L.; Morokuma, K.; Farkas, O.; Foresman, J. B.; Fox, D. J. Gaussian 16 Rev. C.01, Wallingford, CT, 2016.
3. Chamorro, E.; Duque-Norena, M. & Perez, P. A Comparison Between Theoretical and Experimental Models of Electrophilicity and Nucleophilicity *J. Mol. Struct.: THEOCHEM* **896**, 73– 79 (2009).
4. Zhao, Y. & Truhlar, D. G. The M06 suite of density functionals for main group thermochemistry, thermochemical kinetics, noncovalent interactions, excited states, and transition elements: two new functionals and systematic testing of four M06-class functionals and 12 other functionals. *Theor. Chem. Acc.* **120**, 215–241(2008).
5. Clark, T.; Chandrasekhar, J.; Spitznagel, G. W. & Schleyer, P. V. R. Efficient diffuse function-augmented basis sets for anion calculations. III. The 3-21+ G basis set for first-row elements, Li–F. *J. Comput. Chem.* **4**, 294–301 (1983).
6. Hariharan, P. C. & Pople, J. A. The influence of polarization functions on molecular orbital hydrogenation energies. *Theoret. Chim. Acta* **28**, 213–222 (1973).
7. Hehre, W. J.; Ditchfield, R. & Pople, J. A. Self—consistent molecular orbital methods. XII. Further extensions of Gaussian—type basis sets for use in molecular orbital studies of organic molecules. *J. Chem. Phys.* **56**, 2257–2261 (1972).
8. Krishnan, R.; Binkley, J. S.; Seeger, R. & Pople, J. A. Self-consistent molecular orbital methods. XX. A basis set for correlated wave functions. *J. Chem. Phys.* **72**, 650–654 (1980).
9. Marenich, A. V.; Cramer, C. J. & Truhlar, D. G. Universal solvation model based on solute electron density and on a continuum model of the solvent defined by the bulk dielectric constant and atomic surface tensions. *J. Phys. Chem. B* **113**, 6378–6396 (2009).
10. Legault, C. CYLview, 1.0 b, Université de Sherbrooke. Sherbrooke, QC: http://www. cylview. org 2009.
11. Johns, V. K.; Wang, Z.; Li, X. & Liao, Y. Physicochemical Study of a Metastable-State Photoacid. *J. Phys. Chem. A* **117**, 13101–13104 (2013).
